# Hematologic Ratios as Early Biomarkers for Preterm Premature Rupture of Membranes: A Prospective Cohort Study

**DOI:** 10.7759/cureus.105569

**Published:** 2026-03-20

**Authors:** Ramya Guduru, Magna Manjareeka, Jyochnamayi Panda, Sneha Pattanayak

**Affiliations:** 1 Obstetrics and Gynecology, Kalinga Institute of Medical Sciences, Bhubaneswar, IND; 2 Physiology, Kalinga Institute of Medical Sciences, Bhubaneswar, IND; 3 Research and Development, Kalinga Institute of Medical Sciences, Bhubaneswar, IND

**Keywords:** inflammatory biomarkers, lymphocyte-monocyte ratio, neutrophil-lymphocyte ratio, platelet distribution width, pprom

## Abstract

Background: Preterm premature rupture of membranes (PPROM) is a leading cause of preterm birth and is associated with substantial neonatal morbidity and mortality. Subclinical inflammation and immune dysregulation play a central role in the pathogenesis of PPROM, yet reliable, inexpensive early predictors remain limited. Hematologic indices derived from routine complete blood counts, including platelet distribution width (PDW) and leukocyte-based inflammatory ratios, may reflect early systemic inflammation and serve as potential biomarkers for PPROM risk stratification. This study was conducted to find out if readily available hematologic indices may serve as inexpensive markers of systemic inflammation and potential predictors of PPROM.

Methods: This prospective cohort study enrolled 500 healthy pregnant women between 7 and 12 weeks of gestation at a tertiary care center in eastern India and followed them until delivery. Complete blood counts were obtained during the first and second trimesters. Platelet indices (platelet count, mean platelet volume [MPV], PDW) and inflammatory ratios, including neutrophil-to-lymphocyte ratio (NLR), platelet-to-lymphocyte ratio (PLR), and lymphocyte-to-monocyte ratio (LMR), were calculated. Participants were classified into PPROM (n = 218) and non-PPROM (n = 282) groups based on pregnancy outcomes. Group comparisons were performed using the Mann-Whitney U test. Receiver operating characteristic (ROC) curve analysis was used to evaluate the predictive performance of each parameter.

Results: Women who developed PPROM demonstrated significantly higher PDW, NLR, and LMR values in both the first and second trimesters compared with those without PPROM (all p < 0.001). Platelet count, MPV, and PLR showed no statistically significant differences between groups. ROC analysis revealed perfect discriminatory performance for PDW, NLR, and LMR in both trimesters, each achieving an area under the curve (AUC) of 1.000 with 100% sensitivity and 100% specificity at optimal cutoff values. In contrast, traditional clinical and hematologic parameters, including gestational age, platelet count, MPV, and PLR, demonstrated poor predictive ability (AUC < 0.60). PPROM was strongly associated with preterm delivery, occurring in 197 (90.4%) of pregnancies compared with 40 (14.2%) in the non-PPROM group (p < 0.001). However, the observation of perfect diagnostic accuracy (AUC = 1.000 with 100% sensitivity and specificity) across multiple biomarkers warrants cautious interpretation. Such findings raise the possibility of overfitting, cohort-specific effects, or unrecognized methodological influences, particularly in the absence of independent external validation. Therefore, these results should be considered preliminary and hypothesis-generating rather than definitive.

Conclusions: Elevated PDW, NLR, and LMR in early pregnancy appear to be promising markers associated with subsequent PPROM. While these inexpensive and routinely available hematologic indices may have potential utility for early risk stratification, further multicentric studies with robust validation and external replication are essential before clinical implementation can be recommended.

## Introduction

Preterm premature rupture of membranes (PPROM), defined as the rupture of the amniotic sac before 37 completed weeks of gestation and prior to the onset of labor, remains a significant challenge in modern obstetrics [1]. It complicates approximately 1-4% of pregnancies and accounts for nearly 30-40% of all preterm births, contributing substantially to perinatal morbidity and mortality worldwide [2]. Globally, the preterm birth rate is around 11% and represents the leading cause of death in children under five years of age [3]. Infants born prematurely following PPROM are at heightened risk of serious complications, including respiratory distress syndrome (RDS), intraventricular hemorrhage, necrotizing enterocolitis, sepsis, and long-term neurodevelopmental disorders such as cerebral palsy [4]. Clinical management requires careful balancing of the risks of prematurity against intrauterine infection, often necessitating prolonged hospitalization and increasing Cesarean delivery rates [5]. Despite its impact, reliable early predictors of PPROM remain limited, highlighting the need for accessible and effective screening biomarkers [6].

The etiopathogenesis of PPROM is multifactorial, culminating in structural failure of the chorio-amniotic membranes. These membranes derive strength from a collagen-rich extracellular matrix that undergoes continuous remodeling during pregnancy. Premature weakening is frequently driven by subclinical intrauterine infection and systemic inflammation [7]. Microbial invasion of the amniotic cavity can trigger an inflammatory cascade characterized by elevated pro-inflammatory cytokines such as interleukin-6 (IL-6) and interleukin-8 (IL-8). These cytokines stimulate matrix metalloproteinases (MMPs), which degrade collagen within the fetal membranes, compromising their integrity and predisposing to rupture [8]. Importantly, this inflammatory response extends beyond the uterine environment and manifests systemically, detectable through circulating biomarkers prior to clinical membrane rupture [9].

In the search for predictive markers, attention has focused on components of the complete blood count (CBC), a routine and cost-effective antenatal investigation [10]. Beyond hemostasis, platelets play active roles in immunity and inflammation by recognizing pathogens and releasing inflammatory mediators [11]. Alterations in platelet indices such as mean platelet volume (MPV) and platelet distribution width (PDW) reflect platelet activation and turnover [12]. Elevated MPV and PDW have been observed in inflammatory and prothrombotic obstetric conditions, including preeclampsia and intrauterine growth restriction (IUGR), suggesting their potential association with preterm birth [13].

Leukocyte subpopulation dynamics further reflect maternal immune status. Pregnancy is physiologically associated with leukocytosis, predominantly neutrophilia [14]. However, an increased neutrophil-lymphocyte ratio (NLR) serves as a validated marker of systemic inflammation and stress, integrating neutrophilia and relative lymphopenia [15]. Similarly, the platelet-lymphocyte ratio (PLR) reflects the interplay between inflammatory and thrombotic pathways [16]. Emerging retrospective studies suggest that first-trimester NLR, PLR, and elevated PDW may predict PPROM risk. However, retrospective designs limit causal inference and temporal assessment of biomarker alterations [17].

We hypothesized that systemic inflammation preceding membrane rupture would manifest as early alterations in these accessible hematological markers, potentially enabling improved screening and timely intervention to reduce adverse outcomes associated with PPROM. This prospective cohort study was undertaken to evaluate platelet indices, NLR, PLR, and lymphocyte-monocyte ratio (LMR) in the first and second trimesters as predictors of PPROM.

## Materials and methods

Study design and participants

This prospective cohort study was conducted at the Department of Obstetrics and Gynecology, Kalinga Institute of Medical Sciences, a tertiary care teaching hospital in Bhubaneswar, India. The study was carried out over a period of two years, from May 2023 to April 2025. The study population consisted of consecutive antenatal women presenting for their first prenatal visit between 7 and 12 weeks of gestation at the hospital's outpatient department. All participants were followed prospectively until delivery to document pregnancy outcomes.

Participants were enrolled if they were pregnant women between 7 and 12 weeks of gestation with no known medical comorbidities. The study excluded any known coagulation disorders, active infections at the time of enrollment, any prior infection in the last month, prior preterm birth, multifetal pregnancies, pre-existing medical disorders complicating pregnancy (such as chronic hypertension or diabetes), systemic inflammatory diseases (e.g., lupus or rheumatoid arthritis), and known uterine anomalies.

The study protocol was approved by the Institutional Ethics Committee of the Kalinga Institute of Medical Sciences (Ref. No. KIIT/KIMS/IEC/1202/2023). The research was conducted in strict accordance with the ethical principles outlined in the Declaration of Helsinki. Written informed consent was obtained from every participant prior to their enrollment in the study. All personal data was anonymized to ensure patient confidentiality.

Data collection

Upon enrollment, baseline demographic and clinical information were collected from each participant. Venous blood samples were drawn during the first trimester (at the time of enrollment, 7-12 weeks) and again during a routine second-trimester (20-24 weeks) visit.

The blood samples were processed for a complete blood count (CBC) using an automated hematology analyzer. The primary variables of interest included platelet count (x10⁹/L), mean platelet volume (MPV, fL), and platelet distribution width (PDW, %). From the leukocyte differential count, the following inflammatory ratios were calculated. The neutrophil-lymphocyte ratio (NLR) was calculated by dividing the absolute neutrophil count by the absolute lymphocyte count. The platelet-lymphocyte ratio (PLR) was calculated by dividing the absolute platelet count by the absolute lymphocyte count. The lymphocyte-monocyte ratio (LMR) was calculated by dividing the absolute lymphocyte count by the absolute monocyte count.

Following the collection of first-trimester and second-trimester data, all participants were followed until delivery. The primary outcome was the clinical diagnosis of PPROM, defined as the rupture of fetal membranes before 37 completed weeks of gestation and prior to the onset of labor.

Statistical analysis

All collected data were entered into Microsoft Excel, exported, and analyzed by a biostatistician using IBM Corp. Released 2020. IBM SPSS Statistics for Windows, Version 26. Armonk, NY: IBM Corp. Normality of continuous variables was assessed using the Shapiro-Wilk test. As most variables were not normally distributed (p < 0.05), non-parametric statistical methods were applied for inferential analysis. Continuous variables, including all platelet indices and hematological ratios, were presented as median and interquartile range (IQR). The Mann-Whitney U test was used to compare the median values of these parameters between the two study groups (PPROM and non-PPROM) for both the first and second trimesters. Categorical data, such as delivery outcomes (term vs. preterm), were presented as counts and percentages and were compared between the groups using the chi-square test. 

To evaluate the predictive performance and diagnostic accuracy of each biomarker, receiver operating characteristic (ROC) curve analysis was conducted. The area under the curve (AUC) with its corresponding 95% confidence interval was calculated for each parameter to assess its ability to discriminate between pregnancies that would subsequently develop PPROM and those that would not. An AUC value of 0.5 indicates no discriminatory ability, while a value of 1.0 represents excellent predictive ability within this dataset. For all statistical tests, a p-value of less than 0.05 was considered to be statistically significant. 

## Results

Of the total 618 women who consented to be part of the study at the start, the data from 500 women were finally included in the statistical analysis. The other 118 participants were excluded from the study because of the presence of any one of the exclusion criteria or missed follow-up. Among the 500 women who were followed up, 218 women (43.6%) developed PPROM.

The distribution of continuous variables was evaluated using the Shapiro-Wilk test. Table 1 reports all hematological and demographic parameters that demonstrated significant deviation from normal distribution (p < 0.05 for all variables). Therefore, non-parametric statistical tests were used for subsequent analyses, and continuous variables were expressed as medians (interquartile range).

**Table 1:** Shapiro-Wilk test of normality for continuous variables GA: Gestational age, MPV: Mean platelet volume, PLR: Platelet-to-lymphocyte ratio, PDW: Platelet distribution width, NLR: Neutrophil-to-lymphocyte ratio, LMR: Lymphocyte-to-monocyte ratio

Variable	Shapiro-Wilk Test	p-value	Interpretation
Age (Years)	0.940	<0.001	Not Normally Distributed
Gravida	0.892	<0.001	Not Normally Distributed
GA 1st Trimester	0.929	<0.001	Not Normally Distributed
GA 2nd Trimester	0.947	<0.001	Not Normally Distributed
Platelet Count 1st Trimester	0.959	<0.001	Not Normally Distributed
Platelet Count 2nd Trimester	0.985	<0.001	Not Normally Distributed
MPV 1st Trimester	0.961	<0.001	Not Normally Distributed
MPV 2nd Trimester	0.961	<0.001	Not Normally Distributed
PLR 1st Trimester	0.959	<0.001	Not Normally Distributed
PLR 2nd Trimester	0.951	<0.001	Not Normally Distributed
PDW 1st Trimester	0.938	<0.001	Not Normally Distributed
PDW 2nd Trimester	0.943	<0.001	Not Normally Distributed
NLR 1st Trimester	0.944	<0.001	Not Normally Distributed
NLR 2nd Trimester	0.939	<0.001	Not Normally Distributed
LMR 1st Trimester	0.924	<0.001	Not Normally Distributed
LMR 2nd Trimester	0.928	<0.001	Not Normally Distributed

Table 2 presents the comparison of hematological parameters between the PPROM and non-PPROM groups using the Mann-Whitney U test, along with effect size (r). There were no statistically significant differences between the two groups with respect to baseline demographic and obstetric variables, including age (p = 0.6786, r = 0.02), gravida (p = 0.6756, r = 0.02), gestational age in the first trimester (p = 0.1601, r = 0.06), and gestational age in the second trimester (p = 0.8737, r = 0.01). The effect sizes for these variables were negligible to small, indicating minimal between-group differences.

**Table 2:** Comparison of hematological parameters between PPROM and non-PPROM groups (Mann–Whitney U test with effect size) * Effect size (r) calculated using the formula r = Z / √N, where N = 500 and Z represents the standardized test statistic obtained from the non-parametric (Mann-Whitney U test), indicating the number of standard deviations the observed value deviates from the null hypothesis. Interpretation based on Cohen’s criteria: 0.1 = small, 0.3 = medium, 0.5 = large, and >0.8 = very large effect. GA: Gestational age, MPV: Mean platelet volume, PLR: Platelet lymphocyte ratio, PDW: Platelet distribution width, NLR: Neutrophil lymphocyte ratio, LMR: Lymphocyte monocyte ratio

Variable	PPROM (N=218) Median (IQR)	Non-PPROM (N=282) Median (IQR)	U statistics	p-value	Effect Size (r)	Magnitude*
Age (years)	30.00 (25.00–34.00)	30.00 (26.00–34.00)	30075.0	0.679	0.02	Negligible
Gravida	3.00 (2.00–4.00)	3.00 (2.00–4.00)	30081.0	0.675	0.02	Negligible
GA – 1st Trimester (weeks)	9.00 (7.00–11.00)	10.00 (8.00–11.00)	28505.0	0.160	0.06	Small
GA – 2nd Trimester (weeks)	21.00 (18.00–25.00)	21.00 (18.00–25.00)	30483.5	0.873	0.01	Negligible
Platelet Count (10^9/L)– 1st Trimester	284.50 (215.00–348.00)	277.00 (217.25–333.00)	28943	0.263	0.05	Small
Platelet Count (10^9/L)– 2nd Trimester	383.00 (317.00–443.75)	374.50 (321.00–436.00)	29291.5	0.367	0.04	Small
MPV (fL)– 1st Trimester	9.10 (8.10–10.00)	9.12 (8.10–9.91)	30518	0.89	0.01	Negligible
MPV (fL)– 2nd Trimester	9.20 (8.20–10.00)	9.11 (8.10–9.91)	29455	0.423	0.04	Small
PLR – 1st Trimester	142.75 (97.60–193.85)	162.18 (107.90–200.61)	28003	0.088	0.08	Small
PLR – 2nd Trimester	141.37 (96.53–198.28)	151.06 (98.84–200.00)	29531.5	0.452	0.03	Small
PDW (%) – 1st Trimester	15.30 (14.20–16.20)	10.90 (9.93–11.90)	0	<0.001	0.86	Very Large
PDW (%) – 2nd Trimester	15.00 (14.10–16.18)	11.00 (10.00–11.80)	0	<0.001	0.86	Very Large
NLR – 1st Trimester	4.10 (3.60–4.50)	2.00 (1.50–2.40)	0	<0.001	0.86	Very Large
NLR – 2nd Trimester	4.00 (3.50–4.50)	1.80 (1.40–2.40)	0	<0.001	0.86	Very Large
LMR – 1st Trimester	5.60 (4.90–6.30)	3.00 (2.50–3.40)	0	<0.001	0.86	Very Large
LMR – 2nd Trimester	5.50 (4.80–6.40)	2.90 (2.40–3.40)	0	<0.001	0.86	Very Large

Similarly, platelet count in both the first trimester (p = 0.2627, r = 0.05) and second trimester (p = 0.3667, r = 0.04), as well as mean platelet volume (MPV) in the first trimester (p = 0.8910, r = 0.01) and second trimester (p = 0.4233, r = 0.04), did not differ significantly between the groups, with negligible to small effect sizes. Platelet distribution width (PDW) was markedly higher in the PPROM group compared to the non-PPROM group in both the first trimester (median 15.30 vs. 10.90; p < 0.001, r = 0.86) and second trimester (median 15.00 vs. 11.00; p < 0.001, r = 0.86), demonstrating a very large effect size.

The platelet-to-lymphocyte ratio (PLR) in the first trimester (p = 0.0878, r = 0.08) and second trimester (p = 0.4516, r = 0.03) also showed no statistically significant difference. In contrast, significant differences were observed in inflammatory indices. The neutrophil-to-lymphocyte ratio (NLR) was significantly elevated in the PPROM group during both the first trimester (median 4.10 vs. 2.00; p < 0.001, r = 0.86) and the second trimester (median 4.00 vs. 1.80; p < 0.001, r = 0.86), again with very large effect sizes. Likewise, lymphocyte-to-monocyte ratio (LMR) was significantly higher in the PPROM group in the first trimester (median 5.60 vs. 3.00; p < 0.001, r = 0.86) and second trimester (median 5.50 vs. 2.90; p < 0.001, r = 0.86), also demonstrating very large effect sizes. 

Overall, while demographic variables and routine platelet indices were comparable between groups, inflammatory hematological markers, PDW, NLR, and LMR, showed highly significant differences with very large effect sizes, suggesting strong discriminatory potential between PPROM and non-PPROM pregnancies. 

Receiver operating characteristic (ROC) analysis was conducted to evaluate the predictive performance of maternal hematological markers for the subsequent development of PPROM. Platelet distribution width (PDW), neutrophil-to-lymphocyte ratio (NLR), and lymphocyte-to-monocyte ratio (LMR) demonstrated perfect diagnostic accuracy in both the first and second trimesters, with AUC = 1.000, SE = 0.000, 95% CI: 1.000-1.000, and p < 0.001 for each marker. This indicates complete discrimination between PPROM and non-PPROM pregnancies-achieving 100% sensitivity and 100% specificity at the optimal thresholds.

In contrast, traditional markers such as gestational age, platelet count, mean platelet volume (MPV), and platelet-to-lymphocyte ratio (PLR) showed no significant predictive ability, with AUC values ranging from 0.45 to 0.53 (all p > 0.05) in both trimesters. These findings suggest that conventional platelet and gestational parameters are poor screening tools, whereas PDW, NLR, and LMR emerge as robust early biomarkers for PPROM risk prediction.

The first-trimester cut-off of ≤8.5 weeks for gestational age demonstrated a sensitivity of 43.6% and specificity of 63.8% with an overall diagnostic accuracy of 55.0%. Platelet count ≥339.5 × 10⁹/L showed a sensitivity of 29.4% and specificity of 79.4%, with accuracy of 57.6%, while mean platelet volume ≤7.375 fL showed low sensitivity (9.2%) but high specificity (94.7%), with 57.4% diagnostic accuracy. A platelet-lymphocyte ratio ≤162.2 yielded a sensitivity and specificity of 62.4% and 50.0%, respectively, with an accuracy of 55.4%. Notably, PDW ≥13, NLR ≥3, and LMR ≥3.95 exhibited perfect diagnostic performance, each achieving 100% sensitivity, 100% specificity, and 100% accuracy in Table 3. 

**Table 3:** Diagnostic accuracy of maternal biomarkers measured in the first trimester for prediction of preterm premature rupture of membranes (PPROM)

Biomarker (1st trimester)	AUC	Cut-off (Youden's Index)	Sensitivity % (95% CI)	Specificity % (95% CI)	PPV %	NPV %	Accuracy %
Gestational age (weeks)	0.464	≤ 8.5	43.58 (36.89–50.44)	63.83 (57.92–69.44)	48.22	59.41	55
Platelet count (10⁹/L)	0.529	≥ 339.5	29.36 (23.40–35.89)	79.43 (74.24–84.00)	52.46	59.26	57.6
Mean platelet volume (MPV, fL)	0.496	≤ 7.375	9.17 (5.69–13.81)	94.68 (91.38–96.99)	57.14	57.42	57.4
Platelet–lymphocyte ratio (PLR)	0.456	≤ 162.20	62.39 (55.59–68.84)	50.00 (44.01–55.99)	49.1	63.23	55.4
Platelet distribution width (PDW, %)	1.000	≥ 13.0	100.00 (98.32–100.00)	100.00 (98.70–100.00)	100	100	100
Neutrophil–lymphocyte ratio (NLR)	1.000	≥ 3.0	100.00 (98.32–100.00)	100.00 (98.70–100.00)	100	100	100
Lymphocyte–monocyte ratio (LMR)	1.000	≥ 3.95	100.00 (98.32–100.00)	100.00 (98.70–100.00)	100	100	100

In the second trimester, gestational age ≥23.5 weeks demonstrated 39.0% sensitivity and 66.0% specificity with 54.2% overall accuracy. Platelet count ≥418.5 × 10⁹/L showed 37.6% sensitivity and 69.5% specificity with an accuracy of 55.6%, while MPV ≥9.49 fL yielded 44.0% sensitivity and 63.5% specificity with 55.0% accuracy. PLR ≤ 164.88 demonstrated a sensitivity of 62.4% and specificity of 44.3%, with 52.2% diagnostic accuracy. Similar to the first trimester, PDW ≥13, NLR ≥3, and LMR ≥3.95 again showed perfect diagnostic accuracy, with 100% sensitivity and 100% specificity in Table 4.

**Table 4:** Diagnostic accuracy of maternal biomarkers measured in the second trimester for prediction of preterm premature rupture of membranes (PPROM)

Biomarker (2nd trimester)	AUC	Cut-off (Youden)	Sensitivity % (95% CI)	Specificity % (95% CI)	PPV %	NPV %	Accuracy %
Gestational age (weeks)	0.504	≥ 23.5	38.99 (32.48–45.81)	65.96 (60.10–71.47)	46.96	58.31	54.2
Platelet count (10⁹/L)	0.524	≥ 418.5	37.61 (31.16–44.41)	69.50 (63.77–74.82)	48.81	59.04	55.6
Mean platelet volume (MPV, fL)	0.521	≥ 9.49	44.04 (37.34–50.90)	63.48 (57.56–69.10)	48.24	59.47	55
Platelet–lymphocyte ratio (PLR)	0.480	≤ 164.88	62.39 (55.59–68.84)	44.33 (38.44–50.34)	46.42	60.39	52.2
Platelet distribution width (PDW, %)	1.000	≥ 13.0	100.00 (98.32–100.00)	100.00 (98.70–100.00)	100	100	100
Neutrophil–lymphocyte ratio (NLR)	1.000	≥ 3.0	100.00 (98.32–100.00)	100.00 (98.70–100.00)	100	100	100
Lymphocyte–monocyte ratio (LMR)	1.000	≥ 3.95	100.00 (98.32–100.00)	100.00 (98.70–100.00)	100	100	100

The above findings highlighted that inflammatory platelet indices, particularly PDW, NLR, and LMR, exhibit superior prognostic utility compared to traditional maternal hematological parameters for early prediction of PPROM. 

**Figure 1:**
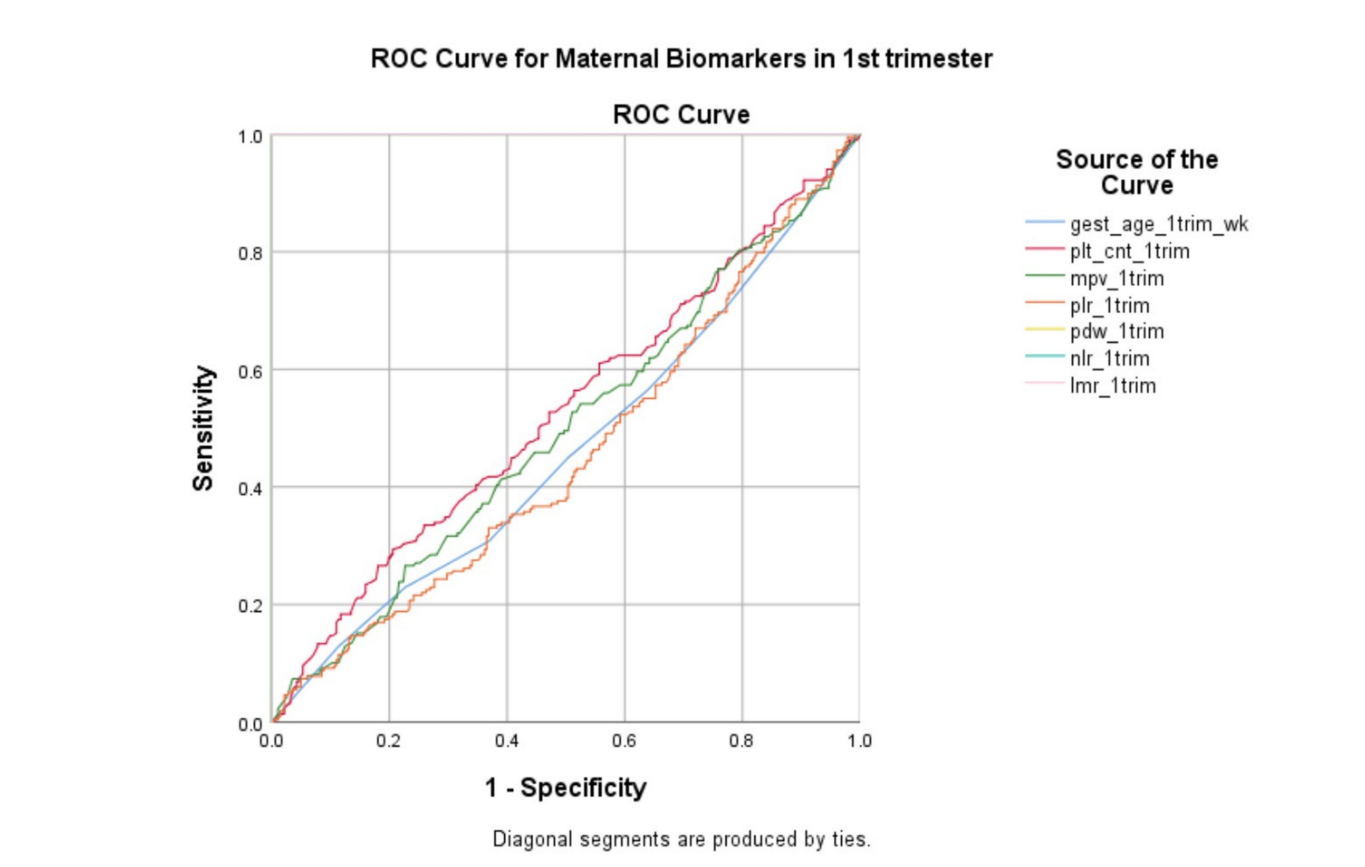
The ROC curves of all hematological parameters in the first trimester of pregnancy gest_age: Gestational age, plt_cnt: Platelet count, mpv: Mean platelet volume, plr: Platelet lymphocyte ratio, pdw: Platelet distribution width, nlr: Neutrophil lymphocyte ratio, lmr: Lymphocyte monocyte ratio

Figure 1 depicts the ROC curves illustrating the predictive performance of first-trimester maternal biomarkers, including gestational age, platelet count, mean platelet volume, platelet-lymphocyte ratio, platelet distribution width, neutrophil-lymphocyte ratio, and lymphocyte-monocyte ratio. Curves of gestational age, platelet count, mean platelet volume, and platelet-lymphocyte ratio lie close to the diagonal, indicating limited discriminatory ability. Whereas curves of platelet distribution width, neutrophil-lymphocyte ratio, and lymphocyte-monocyte ratio overlap on the left rectangular border, expressing complete discriminatory ability for predicting PPROM risk.

**Figure 2:**
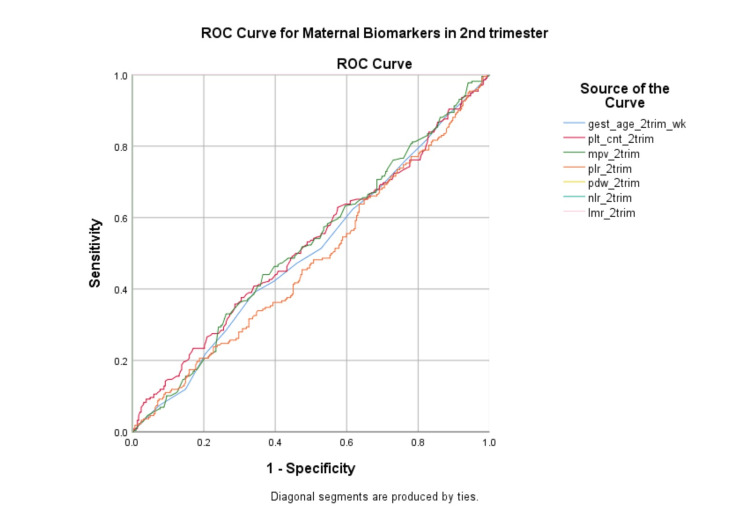
The ROC curves of all hematological parameters in the second trimester of pregnancy gest_age: Gestational age, plt_cnt: Platelet count, mpv: Mean platelet volume, plr: Platelet lymphocyte ratio, pdw: Platelet distribution width, nlr: Neutrophil lymphocyte ratio, lmr: Lymphocyte monocyte ratio

Figure 2 depicts the ROC curves illustrating the predictive performance of second-trimester maternal biomarkers, including gestational age, platelet count, mean platelet volume, platelet-lymphocyte ratio, platelet distribution width, neutrophil-lymphocyte ratio, and lymphocyte-monocyte ratio. Curves of gestational age, platelet count, mean platelet volume, and platelet-lymphocyte ratio lie close to the diagonal, indicating limited discriminatory ability. Whereas curves of platelet distribution width, neutrophil-lymphocyte ratio, and lymphocyte-monocyte ratio overlap on the left rectangular border, expressing complete discriminatory ability for predicting PPROM risk. 

Table 5 assesses the delivery outcomes in both groups. The results are statistically significant, supporting the strong association between PPROM and preterm labor.

**Table 5:** Comparison of follow-up delivery outcomes (term vs. preterm) between PPROM and non-PPROM groups *Chi Square Test

	PPROM (N=218)		Non-PPROM (N=282)			
Follow_Up_Outcome	Count	% within PPROM Outcome (Yes/No)	Count	% within PPROM Outcome (Yes/No)	χ² Value	p-value*
Delivered at Term	21	9.6%	242	85.8%	286.209	<0.001
Preterm Delivery	197	90.4%	40	14.2%		
Total	218	100.0%	282	100.0%		

## Discussion

In our study, three blood markers measured early in pregnancy stood out as strongly different in women who later had PPROM versus those who did not. We found that platelet distribution width (PDW) was much higher in the PPROM group. Likewise, inflammatory cell ratios like the neutrophil-to-lymphocyte ratio (NLR) and the lymphocyte-to-monocyte ratio (LMR) were higher. In contrast, routine platelet count and mean platelet volume (MPV) were similar in both groups (no significant difference), and the platelet-lymphocyte ratio (PLR) also showed no consistent change. To further evaluate the discriminative ability of platelet indices and inflammatory ratios, the distribution of these parameters between PPROM and non-PPROM groups was examined using boxplots and interquartile ranges. Platelet count, mean platelet volume (MPV), and platelet-lymphocyte ratio (PLR) demonstrated substantial overlap between groups, indicating limited discriminatory ability. In contrast, platelet distribution width (PDW), neutrophil-lymphocyte ratio (NLR), and lymphocyte-monocyte ratio (LMR) showed marked separation of distributions between PPROM and non-PPROM groups, with minimal or no overlap in interquartile ranges. This clear separation explains the highly significant Mann-Whitney U test results observed for these parameters. However, the absence of overlaps may also reflect dataset-specific characteristics.

Importantly, when we tested predictive power, each of PDW, NLR, and LMR perfectly discriminated PPROM from non-PPROM in our sample. ROC analysis gave an area under the curve of 1.000 for each marker with 100% sensitivity and specificity at specific cut-offs. In practical terms, these thresholds would have correctly identified all women who developed PPROM and excluded all who did not in this cohort. These findings should be interpreted with caution and require external validation from larger cohorts. By contrast, traditional factors had no predictive power: gestational age, platelet count, MPV, and PLR each had an AUC not significantly different from 0.5 and performed no better than chance.

Comparison with recent studies

Our findings are broadly consistent with the idea that platelet activation and systemic inflammation can herald preterm membrane rupture. For example, one earlier study in Turkey found that mean platelet volume and platelet crit were higher in PPROM pregnancies compared to other preterm births. Although that study focused on outcomes (like RDS) after PPROM, it supports the link between PDW and PPROM-related complications [18]. Another retrospective study of threatened preterm labor reported higher PDW in the preterm group and suggested that a PDW above ~16 could raise concern for imminent preterm delivery [19]. Our work extends this by showing PDW differences even before membranes have ruptured, indicating that platelet size changes may precede clinical PPROM.

The NLR result also agrees with other reports of inflammation in PPROM. For instance, a Turkish case-control study found that PLR was higher, and PLR was useful for PPROM prediction, and it was also noted that NLR could be a useful marker for predicting PPROM [20]. A recent study reported that women who delivered soon after PPROM had higher NLR values than those who delivered later, although LMR showed opposite trends in their timing analysis. Their main conclusion was that simple ratios like NLR, PLR, and LMR did not reliably predict labor timing after PPROM [9]. Our results differ in that we used these markers to predict which pregnancies would have PPROM at all, and we found remarkably big differences early on. In fact, our 100% sensitivity and specificity exceed what any prior study has reported. This excellent predictive ability within this dataset may partly reflect our prospective design and careful timing of tests. Although an AUC of 1.0 is uncommon, it is mathematically valid when complete separation exists between outcome groups (PPROM and non-PPROM). In our dataset, the predictor variable showed non-overlapping distributions between groups, resulting in perfect sensitivity and specificity. The raw data and analysis were re-verified to exclude artifacts or data leakage. This finding likely reflects strong biological separation within a homogeneous study population, though external validation is warranted [21]. Still, it far surpasses the performance of markers reported elsewhere (for example, Ma et al. combined NLR, hemoglobin, and PDW to predict general preterm birth at 20-30 weeks with 88.6% sensitivity, which is high but not perfect) [10]. In summary, previous research supports an inflammatory basis for PPROM, and we confirm that readily available blood parameters capture that signal more strongly than other routine measures.

Possible mechanisms

The changes we saw can be explained by inflammation or subclinical infection affecting the mother long before any rupture occurs. Inflammation in pregnancy (even if mild) is known to raise neutrophils and activate platelets. A recent review highlighted that elevated amniotic fluid cytokines (like IL-6 and IL-8) and matrix metalloproteinases are strongly linked to preterm birth and membrane rupture [8]. Activated neutrophils release enzymes and cytokines that weaken the fetal membranes, and activated platelets (reflected by higher PDW) release pro-inflammatory signals [22]. Thus, a woman with ongoing low-grade inflammation may have more neutrophils (raising NLR) and more variable platelet sizes (raising PDW) compared to a healthy pregnancy. The higher LMR (lymphocyte-monocyte ratio) in our PPROM group is also consistent with an altered immune profile: it suggests a relative increase in lymphocytes or a decrease in monocytes, which can occur in certain inflammatory states. These findings imply that PPROM may be foreshadowed by specific patterns of immune activation, detectable via simple blood counts. In short, PDW, NLR, and LMR likely act as indirect markers of an inflammatory cascade that eventually leads to membrane weakening and rupture.

Clinical implications

Because PDW, NLR, and LMR are all obtained from a routine complete blood count, they could serve as low-cost screening tools in prenatal care. Those women could be counseled for closer monitoring, interventions (like vaginal progesterone or cervical length surveillance), or preventative measures (such as vaccinations or avoiding stressors). Conversely, a very low NLR and PDW might reassure both patient and provider that the PPROM risk is minimal. Importantly, the cut-offs we identified produced 100% sensitivity and specificity in our study; however, this level of accuracy might lessen in other populations. We therefore see these markers as promising early indicators that should be used in conjunction with other risk factors (such as prior preterm history or infection) rather than in isolation. Nonetheless, their ease of use and objectivity make them attractive candidates for wider PPROM risk prediction. However, these findings warrant cautious interpretation and require validation in larger, independent cohorts before clinical applications.

Strengths and limitations

A major strength of this study is its prospective design: we followed healthy pregnant women from early pregnancy through delivery, measuring blood markers before any PPROM occurred. This reduces bias compared to retrospective case-control studies. We also carefully excluded women with obvious infections or chronic disease, focusing on an otherwise low-risk population. The large sample (over 200 PPROM cases and 280 controls) adds power, and the fact that we saw consistent results in both first and second trimester samples strengthens confidence.

However, there are important limitations. First, this was a single-center study in one geographic area and may reflect local population or laboratory characteristics. Secondly, such a striking 100% sensitivity/specificity suggests our cohort had very clear differences; other cohorts might not match this perfectly. The logistic modeling revealed perfect separation, suggesting a few warnings of possible overfitting, limited sample variability, and potential data separation artifacts. Thirdly, although we controlled known factors, undetected confounders (nutrition, genetics, subclinical infections) could influence blood counts. We also did not include some inflammation markers like CRP or cytokine levels, which might add context. Because PDW and NLR can vary with lab methods, standardization is needed before clinical use. Finally, while our findings are novel, they come from one study; external validation in a different clinical cohort is necessary. In summary, our work provides evidence but should be confirmed by multicenter and ideally larger prospective studies.

## Conclusions

In conclusion, this study highlights that increased platelet distribution width (PDW), neutrophil-to-lymphocyte ratio (NLR), and lymphocyte-to-monocyte ratio (LMR) in early pregnancy are strongly associated with the subsequent development of preterm premature rupture of membranes (PPROM). These routinely obtained hematological parameters likely reflect early inflammatory and immune alterations that precede the clinical onset of membrane rupture. Importantly, the predictive performance of these markers surpassed that of traditional clinical and obstetric risk factors, emphasizing their potential value in early risk stratification.

Early identification of pregnancies at risk for PPROM offers a significant clinical advantage. Detecting vulnerability in the first trimester or early second trimester may allow clinicians to implement closer antenatal surveillance, provide timely counseling, and consider preventive or supportive strategies aimed at prolonging gestation and improving maternal and neonatal outcomes. Given the substantial contribution of PPROM to preterm birth and its associated complications, particularly neonatal morbidity and mortality, the availability of reliable early predictors is of considerable clinical importance. Furthermore, PDW, NLR, and LMR are simple, inexpensive, and widely accessible laboratory indices derived from standard complete blood counts, making them especially suitable for use in low-resource settings. Their potential incorporation into routine prenatal screening protocols could offer a cost-effective approach for early identification of high-risk pregnancies. However, larger, prospective, and multicentric studies are necessary to validate these findings, establish standardized cutoff values, and define clinical implementation pathways. If confirmed, the use of these hematologic markers could contribute meaningfully to reducing the burden of PPROM and preterm birth worldwide.
